# Did the house mouse (*Mus musculus* L.) shape the evolutionary trajectory of wheat (*Triticum aestivum* L.)?

**DOI:** 10.1002/ece3.724

**Published:** 2013-08-28

**Authors:** C F Morris, E P Fuerst, B S Beecher, D J Mclean, C P James, H W Geng

**Affiliations:** 1 USDA-ARS Western Wheat Quality LaboratoryPullman, Washington; 2 Department of Crop and Soil Sciences, Washington State UniversityPullman, Washington; 3 Center for Reproductive Biology and the Department of Animal Science, Washington State UniversityPullman, Washington

**Keywords:** Grain hardness, house mouse, kernel texture, puroindolines, wheat

## Abstract

Wheat (*Triticum aestivum* L.) is one of the most successful domesticated plant species in the world. The majority of wheat carries mutations in the *Puroindoline* genes that result in a hard kernel phenotype. An evolutionary explanation, or selective advantage, for the spread and persistence of these hard kernel mutations has yet to be established. Here, we demonstrate that the house mouse (*Mus musculus* L.) exerts a pronounced feeding preference for soft over hard kernels. When allele frequencies ranged from 0.5 to 0.009, mouse predation increased the hard allele frequency as much as 10-fold. Studies involving a single hard kernel mixed with ∼1000 soft kernels failed to recover the mutant kernel. Nevertheless, the study clearly demonstrates that the house mouse could have played a role in the evolution of wheat, and therefore the cultural trajectory of humankind.

## Introduction

Wheat (*Triticum aestivum* L.) is one of the most successful domesticated plant species in the world, and is grown on 220 × 10^6^ ha (15% of the world's total arable land) with an estimated grain production of 700 million metric tons (Food and Agriculture Organization of the United Nations). In the major exporting nations, wheat grain production is segregated based on the hardness or the “texture” of the kernel (caryopsis), that is, whether the texture is “hard” or “soft” (Morris and Rose 1996). In Argentina, Australia, Canada, and the U.S., the majority of wheat production comes from genetically hard kernel varieties. As described following, this is enigmatic as wheat initially was soft and has accumulated “hardness” mutations. The “Why” and “How” is the focus of this paper.

Wheat is an allohexaploid (2*n* = 6*x* = 42, BBAADD) with three homoeologous sets of seven haploid chromosomes. *T. aestivum* arose through the hybridization of the tetraploid *T. turgidum* subsp. *dicoccoides* and a wild diploid relative, *Aegilops tauschii* Coss. Apparently, this hybridization occurred a very few times (Zohary and Hopf 2000; Massa and Morris 2006) in the fields of Neolithic farmers of the Fertile Crescent. Regardless of the number of discrete hybridizations, *T. aestivum* was deduced to be initially monomorphic for the *Puroindoline a* and *Puroindoline b* genes, which comprise the *Hardness* locus on the distal end of chromosome 5D short arm and make kernels soft (Morris 2002; Massa et al. 2004, 2007; Bhave and Morris 2008a,b; Charles et al. 2009). The tetraploid parent, *T. turgidum*, lacks A– and B– genome *Puroindoline* genes due to illegitimate recombination during its formation (Chantret et al. 2005), and hence its kernels are very hard. The hybridization with *Ae. tauschii* restored the *Puroindoline a* and *Puroindoline b* genes, and with them, soft kernel texture to hexaploid wheat.

The “hard” kernel phenotype of *T. aestivum* is now recognized to result from mutations in either *Puroindoline a* (*Pina*) or *Puroindoline b* (*Pinb*). The first of these mutations to be discovered was a SNP in *Pinb*, causing a change from glycine to serine at position 46 of the translated protein (Giroux and Morris 1997), and a considerably harder kernel (due to a reduction in *Puroindoline* softening effect). The second mutation involved a large (15,380 bp) deletion in *Pina* which prevents translation (Giroux and Morris 1998; Morris and Bhave 2008). Since these initial reports, more than 20 additional mutations in *Puroindolines* have been reported (Bhave and Morris 2008a,b; Morris and Bhave 2008). All are associated with a change in kernel texture phenotype from soft to hard.

The establishment and persistence of these puroindoline mutations in wheat (and the hard kernel phenotype that results) is enigmatic, involving the advent and expansion of sedentary agriculture, and the culinary and anthropogenic interaction of perhaps several organisms. Although the role of puroindolines has been suggested as antipathogenic based on transgenic experiments in rice (Krishnamurthy et al. 2001), tetraploid wheat (Luo et al. 2008), maize (Zhang et al. 2011), and hexaploid wheat (Kim et al. 2012), and an in vitro study using puroindoline-derived peptides (Phillips et al. 2011), there has been no explanation or demonstration as to the evolutionary advantage of puroindolines (or lack thereof) in agricultural ecosystems. For example, although apparently many wild members of the Triticeae (perhaps all diploids) possess puroindolines or puroindoline homologs, the tetraploid wheat *T. turgidum* does not, and appears to be at no particular disadvantage for pest resistance in the wild (e.g., wild Emmer) or in agricultural settings (e.g., durum wheat). Similarly, the ascension of hard kernel “bread” wheat in modern agriculture suggests that the “soft” wild-type puroindoline genes are not absolutely advantageous.

In related research (Morris et al. 2012), we observed that the house mouse (*Mus musculus* L.) showed a marked (up to fivefold) preference for soft wheat kernels over hard, that is, hard wheat kernels were avoided and soft were consumed. The house mouse has undergone a long evolutionary relationship with humans, ostensibly since a commensal relationship developed in sedentary agricultural settings (permanent, year-round structures for habitation and grain storage). This led us to formulate the hypothesis that the house mouse, due to feeding preferences, exerted phenotypic selection for hard kernel texture in wheat, thereby increasing the frequency of the hard mutant *Puroindoline* allele at the *Hardness* locus. The theoretical extension of this question asks if the house mouse shaped the evolutionary trajectory of wheat by facilitating the persistence and expansion of hard texture mutations, that is, a case of soft kernel predation versus hard kernel avoidance. Our results indicate that indeed the house mouse can exert dramatic selection pressure on wheat kernel texture, and support the possibility that hardness mutations persisted as “predation defense” genes in prehistoric agriculture. That these mutations now predominate in world wheat production has cultural implications for humankind.

## Material and Methods

### Plant material

Hard (PI644080, *Pina-D1b*) and soft (PI566596, *Pina-D1a*) back-cross seven (BC_7_) near-isogenic lines of the soft white cv. Alpowa (Morris and King 2008) were used to eliminate differences due to different genetic backgrounds. The lines are, in theory, 99.6% identical, and are likely even more similar than this estimate as the donor of the hardness mutation, cv. ID377s, shares substantial parental relatedness, and agronomic and pest resistance gene linkage blocks in common with Alpowa. Of note, the *Hardness* locus resides on a very small piece of chromosome with apparently no linkage disequilibrium (Gill et al. 1996; Morris and Beecher 2012). Both hard and soft grain lots were sieved (U.S. No. 7 Standard Testing Sieve) to eliminate small kernels and to obtain a more consistent average kernel weight (∼40 mg). Kernels were plump and free of disease and insect damage.

### Mice

All animal experiments were approved by the Washington State University Institutional Animal Care and Use Committee (ASAF#03964-001). Female C57Bl/6 mice were randomly selected at 6 weeks of age from a breeding colony originating from the Jackson Laboratory (Bar Harbor, ME; mouse stock number 000664). The mice had been housed in their boxes and had been consuming various varieties of wheat grain for about 9 months prior to the start of the study. Mice were provided standard chow (Harlan 2018, Indianapolis, IN; 180 g kg^−1^ crude protein) and water ad libitum. Environmental conditions were 14-h light:10-h dark, temperature 20–22°C, and 20–30% relative humidity. Cages (28 cm length × 17 cm width × 11 cm height) were filled with ca. 1.8 L of paper bedding with an average size of 1 cm (Harlan 70-L).

### Feeding trials

For all trials, wheat kernels were introduced to the mice in stainless steel feeders. Trial 1 used equal 50:50 blends by weight of soft and hard kernel texture classes which were prepared using the established system of marking each kernel of the two classes with a tiny dot with one of two different colors of ink (Morris et al. 2012). Repeated trials have demonstrated no detectable effect of the dot or the color on feeding preference (Morris et al. 2012; Fuerst et al. 2013; data not shown). A total of 9.0 g (4.5 g each texture class) of kernels were provided to each of 14 mice. After 24 h all the bedding and contents of the cage, including any uneaten kernels were recovered, the kernels were manually isolated, sorted according to color marking, and weighed. New bedding was immediately introduced, the mouse was returned to its cage, and a new sample of wheat was introduced. Consumption was determined by subtraction. The experiment was repeated for a total of four consecutive 24-h periods. Data were analyzed using mice as replicates, and days as repeated measures (see Morris et al. 2012; Fuerst et al. 2013).

In Trial 2, the mice were randomly assigned to one of two groups (seven mice in each group). For the first 24-h period, group one received a single hard kernel mixed with 109 soft kernels; group two received 11 hard kernels mixed with 99 soft kernels (for approximately 1% and 10% frequencies of hard kernels, respectively). In Trial 2 no ink marking was used. As before, uneaten kernels were recovered after 24 h. In this trial, however, individual kernels were separated from some partially eaten kernels; the whole kernels were retained for planting, the partial kernels were retained for direct DNA analysis. On the second day of Trial 2, mixtures were increased to 2 hard+128 soft or 13 hard+117 soft kernels. On the third and fourth days of the trial, mixtures were adjusted to accommodate total consumption of individual mice, and included hard+soft blends of 2 + 128, 2 + 148, 13 + 117, and 15 + 135. In total, over the 4 days, 300 hard and 5090 soft kernels were given to the mice.

Due to limited greenhouse resources, we sought to limit the number/amount of kernels/grain that would require analysis. Consequently, we attempted to only slightly exceed the 24-h consumption of grain for each mouse. Generally, we succeeded but in some instances no wheat kernels were remaining after 24 h, and certainly a variable amount was recovered that was related to the individual consumption of each mouse. Additionally, mice varied in the extent to which they “kibbled” wheat, that is, consumed only part of the kernel. However, kibbling was always related to a preferred consumption of the germ and a variable amount of the endosperm. The total number of kernels was adjusted for individual mice consumption history, attempting to keep an approximate ratio of either 1:10 or 1:100.

Trial 3 involved giving each of the 14 mice 40 g of soft wheat with a single hard kernel. Uneaten kernels were recovered after 3 days. All were retained for planting. For two mice, no kernels were recovered (they ate all the grain).

### Plant culture and DNA analysis

Uneaten kernels were planted in a glasshouse into 2.5-cm diameter plastic cones (height 3.75 cm, arrayed on 4.5 cm centers in racks) at the Washington State University Plant Growth Facility. The bottoms of the cones were immersed in water and the artificial growth media (a peat-based SunShine mix product LC1; Sun Gro Horticulture Canada Ltd., Vancouver, British Columbia) was watered by capillary action. The greenhouse environment was set at 21–24°C day, 15–18°C night. A 16-h photoperiod was used and supplemented with 350 μE of light using 1000-W high pressure sodium lamps. Nutrients were provided weekly in irrigation water (a peat lite special 20–10–20, N–P–K, mixture delivered at 100 ppm N). At about the three to four-leaf stage, ∼10 cm of leaf tissue was collected, freeze dried, and DNA isolated after the method of Riede and Anderson (1996). Plants were grown to maturity, harvested, and kernel texture class was determined using the Single Kernel Characterization System 4100 (SKCS) (Perten Instruments, Springfield, IL). The SKCS weighs and crushes individual kernels and converts the force–crush profile to a unitless hardness index (Morris 2002). Essentially, all the kernels available from each plant were run through the machine which destructively crushes them. These phenotypic data were used to corroborate the DNA analysis, and given the large number of plants, to check for clerical errors.

A number of partial kernels were recovered from the bedding having, without exception, a variable amount of the germ end consumed (see fig. 4 of Morris et al. 2012). The amount of the kernel that was consumed varied from essentially only the germ being eaten to only a small amount of the distal “brush” end remaining. A number of these partial kernels wherein half or more of the kernel was remaining was used to isolate DNA directly using the method of Lagudah et al. (1991).

*Puroindoline a* alleles (*Pina-D1a*, soft wild-type, and *Pina-D1b*, hard mutant) were assessed for all DNA samples using PCR and allele-specific primers after the method of Geng et al. (2012) (Table 1). PCR reactions were performed in an MJ Research PTCB200 thermal cycler in a total volume of 25 μL including 250 μmol/L of each dNTPs, 10 pmol of each primer, 100 ng of gDNA, 1× reaction buffer (50 mmol of KCl, 10 mmol of Tris-Cl, 1.5 μmol/L of MgCl_2_, pH 8.4), and 1 unit of *Taq* DNA polymerase (Promega, Madison, WI). PCR conditions were 94°C for 5 min, followed by 45 cycles of 94°C for 50 sec, 60°C for 50 sec, and 72°C for 1 min, with a final extension of 72°C for 10 min. The PCR products were separated by electrophoresis in a 1.5% (w/v) agarose gels. The bands were stained with ethidium bromide and visualized using UV light.

**Table 1:** PCR primers used in generating *Puroindoline a-D1* allele products in wheat

Allele	Forward primer	Reverse primer	PCR annealing temperature	Fragment size
*Pina-D1a*	TCACCAGTAATAGCCAATAGTG	ATGAAGGCCCTCTTCCTCA	60°C	447 bp
*Pina-D1b*	ACAACCGCACACAGAAATCG	CAATGGGCGCCACTATAACA	60°C	326 bp

### Statistical analysis

For Trial 1, consumption of hard and soft kernels were compared on a gram weight basis using Student's *t*-test with *H*_o_ = no difference in relative consumption. Mice were replicates and days were repeated measures. Data were analyzed by analysis of variance using the following SAS code (SAS v. 9.3; SAS Institute, Cary, NC): proc mixed; model consump = day diet day*diet; random mouse mouse*diet; repeated/subject = mouse*diet type = AR(1); lsmeans diet/adjust = tukey pdiff. The code specifies “consump” equal to grams of each wheat variety consumed, “day” equal to days 1, 2, 3, and 4, “diet” equal to the hard and soft variety class variable, and “mouse” equal to the replicate mice, “type” is the autoregression 1 covariance structure option. *P*-values for the LS mean differences were computed using Tukey's “honestly significant difference” adjustment of Student's *t* distribution. χ^2^ analysis was conducted with 1 degree of freedom to contrast the frequency of hard kernels provided versus hard kernels recovered.

## Results

Trial 1 demonstrated that mice exert a strong consumption preference for soft kernels, avoiding hard textured kernels when at the beginning of the trial they would encounter each type with equal frequency (50:50 blend). Mean consumption was 0.86 g hard, 2.61 g soft (|*t*| = 14.1, *P* = 2.2 × 10^−9^), indicating a threefold preference for soft. As the two grain lots were near-isogenic for kernel texture (exploiting the marked phenotypic difference ascribed to the *Hardness* locus, in this case the null mutation in *Puroindoline a, Pina-D1b*), preference was, for all intents, directly attributable to texture per se and not to other taste, olfactory or tactile traits, nor to differences in kernel size, bran color, etc. which might be related to different genetic backgrounds.

Trial 2 aimed to establish the extent to which the house mouse, conceptually living in a commensal relationship with some access to grain stores, could exert a selection for kernel texture within a mixed population of soft and hard kernels such that the allele frequency would shift. The premise was that hard, mutant kernels would initially be in the minority. The mouse cohort was randomly divided into two groups, which were assigned the two approximate ratios of ca. 1:10 or 1:100, hard:soft. Even though we have repeatedly demonstrated that our ink marking system exerts no detectable consumption/selective influence, no marking was employed in Trial 2. Instead, an analysis *a posteriori* of the DNA of each individual kernel (grown as a plant to maturity) was analyzed by exploiting the underlying genetic basis (i.e., *Puroindoline* haplotype) of kernel texture phenotype.

A total of 517 kernels were recovered and planted. From these, 442 seedlings were established. Without visually inspecting each kernel using magnification, some were no doubt damaged from partial feeding and did not germinate or emerge. Table 2 presents the genotype analysis of the resultant plants using allele-specific PCR for *Pina-D1*. For χ^2^ analysis, the total number of kernels per ratio was used, for example for the 1:109 ratio which was fed to seven mice, the “expected” values in the χ^2^ were 7 and 763, respectively, whereas the “observed” were 1 and 9. This analysis shows that remarkably, the house mouse was able to discern (and therefore select) the preferred soft kernel texture (and by inference avoid the hard kernel) when the proportion of hard kernels was initially in the range of 0.9% to 10%. After what could be termed “allele selection” by the mice, the proportion of hard kernels ranged from 0 to 53%, averaging 31% overall.

**Table 2:** Consumption of mixtures of hard (*Pina-D1b*) and soft (*Pina-D1a*) wheat kernels by the house mouse (Trial 2), and χ^2^ analysis of allele frequency shift

Day(s) of the trial	Ratio provided (hard:soft)	Frequency of hard (%)	Number of mice	Kernels recovered (whole)	Plants analyzed by DNA (hard:soft)	Frequency of hard (%)	χ^2^
1	1:109	0.91	7	13	1:9	10	750.3
1	11:109	9.2	7	28	7:10	41	806.8
2	2:128	1.5	7	24	0:20	0	870.5
2	13:117	10.0	7	79	31:29	52	801.6
3, 4	2:128	1.5	3	99	12:82	13	243.5
3, 4	13:117	10.0	4	149	51:88	37	308.6
3, 4	2:148	1.3	4	56	8:43	16	509.1
3, 4	15:135	10.0	3	69	27:24	53	365.6
Total	300:5090	5.57	–	517	137:305	31	4587

Individual mice show a variable propensity for “kibbling” in the sense that a kernel is selected, only partially consumed, and then discarded. As far as we have observed, this behavior always begins by eating the germ. Morris et al. (2012) provide a scanning electron micrograph image of this phenomenon. With some kernels, consumption ends with the germ, in others only a small portion of the distal brush end remains. It would seem that the mice clearly prefer the germ, and whether this is due to some dietary cue (the germ is rich in lipid and protein, ca. 25% each) (Hoseney 1994), or whether, again, it is simply softer and more palatable is unknown. Alternatively, it may be that the “brush” which is comprised of trichomes is a deterrent. Table 3 presents the results of a total of 46 such partially eaten kernels collected and pooled on each of the 4 days of Trial 2. The frequencies of hard kernels ranged from 58% to 100%, with an overall average of 80% (χ^2^ = 5303). These results would seem to clearly indicate that as an individual kernel was being eaten, once past the germ, the harder endosperm may have dissuaded further consumption and the kernel was discarded. However, having consumed the germ, these partially eaten kernels would not contribute to future generations, and thus could play no role in allele frequency shifts and evolution.

**Table 3:** Number of partially eaten wheat kernels and their *Puroindoline* genotype as determined by PCR analysis of *Pina-D1a* (soft) and of *Pina-D1b* (hard) alleles, and the frequency of hard kernels present

Day of the trial	No. of kernels	No. of kernels with *Pina-D1a*	No. of kernels with *Pina-D1b*	Frequency of hard kernels (%)
1	6	2	4	67
2	18	2	16	89
3	10	0	10	100
4	12	5	7	58
Total	46	9	37	80

**Figure 1:**
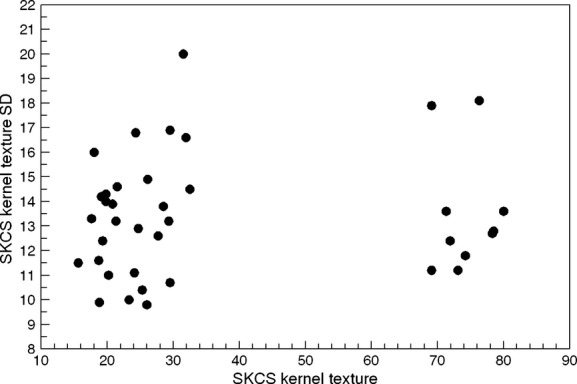
SKCS wheat kernel texture phenotype of selected plants grown from uneaten kernels from Trial 2. Hard and soft kernel mixtures were given to house mice; uneaten kernels were recovered and grown to maturity in a glasshouse. The ordinate is the standard deviation (SD) of the texture of kernels for each plant. “Hard” plants with the mutant *Pina-D1b* allele are to the right (>60), “soft” wild-type *Pina-D1a* plants are to the left (<40).

As noted above, the recovered whole kernels were grown to seedlings, a portion of their leaf tissue was harvested for DNA extraction, and then they were grown to maturity and harvested. To corroborate the DNA analysis (Table 2) and to check for record keeping errors on such a large population, the kernels were analyzed using the SKCS. Figure 1 shows two key features, first, that the expression of the soft and hard alleles, *Pina-D1a* and *Pina-D1b,* exert a pronounced effect on kernel texture phenotype, and second, that all plants could be unambiguously classified phenotypically according to *Hardness* class. The results corroborated the genotype results.

Trial 3 sought to examine the selection for hard kernels when present at very low frequencies, about 1:1000. For two mice, no kernels were recovered (they ate all the grain). Nevertheless, in total, 931 uneaten kernels were recovered and planted. Of these, 859 produced both a seedling and PCR genotype results. Based on PCR, no hard kernels were present. In theory, all 14 hard kernels could have been avoided by the mice and therefore recovered. On the other hand, if no selection occurred (i.e., the ratio remained 1:1000), then the population of uneaten kernels (*n* = 859) would have been insufficient to have likely recovered a single hard kernel.

## Discussion

The factors that result in natural selection can take many forms. The relationships among humans, wheat, and the house mouse present an intriguing scenario for selection and evolution. To begin with, bread wheat (*T. aestivum*) does not persist in the wild without the aid of humans, as its tough rachis and free-threshing glumes prevent competitive seed dispersal (Zohary and Hopf 2000; Simons et al. 2006). Second, an enigmatic feature of much of the wheat grown throughout the globe is that it carries mutations in the *Puroindoline* genes that result in hard kernels. Although one might suggest that “hard wheats are good for bread” (e.g., Morris and Rose 1996), this is largely an anthropological artifact of the last hundred years or so and involves plant breeding, and modern milling techniques to produce refined white flour.

Based on previous observations (Morris et al. 2012), we predicted that the hard kernel phenotype may have discouraged consumption (predation) by the house mouse, thus providing a selective advantage for survival. In ancient times, selection could have occurred in the field at the time of sowing, however, within a matter of hours the widely scattered seed would have been mostly buried, imbibed water, and texture differences would have been lost. Similarly, at maturity, the grain would only express distinct differences in kernel texture once the moisture content had fallen to a level where harvest and storage would proceed. Of further note, the house mouse lives a commensal relationship with humans and would have had near year-round access to grain stores, some of which would necessarily be used for seeding the following year.

At high hard allele frequencies, that is, at 50:50 blends, we observed a pronounced selection against hard kernels (see above). Hard allele frequencies shifted from 0.5 to an average of 0.75. Clearly a caveat to this result is the total amount of grain available at the beginning and then during the trial. Nevertheless, one can envisage that within a limited number of planting/harvesting cycles, mouse predation would indeed shift the population of a theoretical landrace to the hard allele. Once fixed, there would be little opportunity for the *Puroindoline* gene to evolve back to the soft phenotype.

As hard kernels arose by *Puroindoline* gene mutation, their frequency would initially be quite low. Hillman and Davies (1990) used a mutation rate of 10^−6^ for their study of the free-threshing domestication character of wheat. In this regard, it is noteworthy that the puroindolines are nearly unique among genetic systems in hexaploid wheat in that they are represented as a single compound locus and hence behave as a diploid-like character. Estimations of the time required to fix the hard kernel trait in a landrace is well beyond the scope of this paper. However, if we conservatively assume a very low selection coefficient (e.g., 0.05, table 3 of Hillman and Davies, 1990) the allele frequency could still reach 99% in a few centuries.

A mutation rate of 10^−6^ would require, experimentally, a single hard kernel among 10^6^ soft kernels. In our case, about 40 kg of grain. In Trial 3, we set the allele frequency at 10^−3^, but failed to recover any hard kernels. In retrospect the experiment was likely limited by the large quantities of grain that would be necessary, as well as the (probable) need of invoking recurrent selection over several planting/harvesting crop cycles. When we tested our hypothesis using approximately 1% and 10% hard kernel allele frequencies, results were often dramatic (Table 2). In two situations where the beginning frequency was about 1%, a more than 10-fold “enrichment” of the hard *Pina-D1b* allele was observed.

The *Puroindolines* may play a role in biotic (pathogen) plant defense (Krishnamurthy et al. 2001; Luo et al. 2008; Zhang et al. 2011; Kim et al. 2012). However, no convincing role in wheat has been demonstrated in natural ecological or agricultural settings. In this regard, near-isogenic lines which share a very high degree of genetic identity, save the differences at the *Hardness* locus, would control for other confounding factors. Other biotic factors may also be involved. Harlan et al. (1973) state that, “Very hard vitreous seeds store better and suffer less insect damage in the wet tropics than soft chalky seeds. Tendencies in this direction are notable in maize and sorghum.” Whether this statement would apply to wheat in the arid “Fertile Crescent” area of Southwest Asia is unknown.

## Conclusion

An explanation as to the selection of hard kernel mutations in *T. aestivum* has not been established. Here, we demonstrate that the house mouse (*M. musculus*) exerts a pronounced feeding preference for soft over hard kernels. At beginning allele frequencies ranging from 0.5 to 0.009, mouse predation shifted the hard allele frequency as much as 10-fold. The study clearly demonstrates that the house mouse could have played a role in the evolution of wheat, and therefore influenced the cultural trajectory of humankind.
